# Contributors to Neighbour Noise Annoyance

**DOI:** 10.3390/ijerph18158098

**Published:** 2021-07-30

**Authors:** Sarah L. Benz, Julia Kuhlmann, Dirk Schreckenberg, Jördis Wothge

**Affiliations:** 1 ZEUS GmbH, Centre for Applied Psychology, Environmental and Social Research, 58093 Hagen, Germany; kuhlmann@zeusgmbh.de (J.K.); schreckenberg@zeusgmbh.de (D.S.); 2 German Environment Agency, Section Noise Abatement of Industrial Plants and Products, Noise Impact, 06844 Dessau-Roßlau, Germany; joerdis.wothge@uba.de

**Keywords:** neighbour noise annoyance, residential satisfaction, relation to neighbours, satisfaction with neighbourhood

## Abstract

Noise from neighbours has been shown to be one of the most noise annoying sources in Germany, but research on the influencing factors for the annoyance ratings is scarce. Therefore, we investigated whether different personal and contextual (social, physical) factors contribute to neighbour noise annoyance to better understand the neighbour noise annoyance situation. A population-representative survey in four areas in Germany was conducted, with each area further stratified according to their density of agglomeration (inner city, urban outskirt, rural area). Randomly selected residents from each area were invited by mail to participate in the study, either online or via a paper–pencil mode. Noise annoyance was assessed for different noise sources (e.g., neighbourhood, road, railway, aircrafts, different types of industry). In total, 1973 questionnaires were completed. We identified several factors to be predictive of neighbour noise annoyance: satisfaction with the neighbourhood, relationship with neighbours, residential satisfaction, noise sensitivity, and density of agglomeration for people living in the inner city in comparison to rural areas. Particularly, social aspects such as the relationship with neighbours and satisfaction with the neighbourhood have been shown to affect neighbour noise annoyance.

## 1. Introduction

In many countries, there has been a growing trend towards urbanization in the past decades; i.e., a growing number of people live in cities. Cities are becoming increasingly densely populated; i.e., a higher number of people live in spatial proximity to each other. Further, facilities to meet people’s daily needs are also being opened in these densely populated areas. Thus, there are more potential noise sources present in people’s neighbourhoods. Most recently, it has been shown that this trend has shifted in Germany, with more residents moving from cities to surrounding lower density regions [1]. However, cities remain highly dense and, for reasons of sustainability, policy prefers the densification of urban areas [2].

To date, extensive research has been conducted on the effects of environmental noise with an emphasis on traffic noise showing adverse health effects on humans; e.g., WHO Environmental Noise Guidelines [3]. However, living close together creates new challenges beyond traffic noise, one of which is neighbour noise. The German Environment Agency defines neighbour noise as follows: “Noises caused by activities of private persons in the neighbourhood that are disruptive or annoying are referred to as neighbour noise. This includes, for example, loudly tuned televisions, a party, home improvement work in the home or garden, or the operation of vehicles or lawn mowers on private property.” (see [4], translated by the authors). Various studies have investigated the relations between neighbour noise and different health outcomes, and neighbour noise annoyance was shown to be associated with impaired mental health [5,6,7,8,9,10], poor general health status [11], and poor physical health [10,12].

In a study on the relationship between annoyance due to noise from neighbours and health outcomes with people living in multi-storey buildings, an association was found with mental health and with stress: it was observed that people who reported higher levels of neighbour noise annoyance were more likely to have poor mental health and experience higher levels of stress [5]. In a postal study on the influence of the physical and social environment on mental health, dissatisfaction with neighbour noise was further associated with an increase in the risk of poor mental health [9], even when adjusted for other factors related to mental health. In line with this, in a population-based prospective cohort study, it was investigated whether noise annoyance resulting from/caused by different noise sources predicts mental distress five years later [6]. The results indicated that neighbour noise annoyance was predictive of depression, anxiety, and sleep disturbance. High annoyance due to neighbour noise was further observed to be associated with higher odds of suffering from different health symptoms such as pain in different body parts, headache, and sleeping problems [12]. In the European LARES study (Large Analysis and Review of European Housing and Health Status), which was conducted in different European countries, effects of annoyance and both physical and mental health outcomes were observed [10]: in people reporting severe annoyance due to neighbour noise, risks were increased for hypertension, depression, and migraine.

In a study focusing on a particular source of neighbour noise, Park studied the effects of floor impact noise on psychophysiological responses [13]; i.e., annoyance due to and physiological reactions to floor impact noise in multi-storey buildings. Significant effects were observed with increasing noise exposure on electrodermal activity, heart rates, and respiration rates. Annoyance ratings were further correlated with the noticeability of the floor impact noise and were dependent on noise level.

In [10], the authors state that neighbour noise-induced annoyance seems to be an underestimated risk and can contribute to the risk of other health effects. Noise annoyance as a stress response is embodied in cognitive, affective, and behavioural dimensions and can be described as “a relation between an acoustic situation and a person who is forced by noise to do things he/she does not want to do, who cognitively and emotionally evaluates this situation and feels partly helpless” [14]. Neighbour noise annoyance is especially problematic, as it affects many people, and research suggests it to be associated with other health outcomes: according to a biennial survey on environmental awareness by the German Environment Agency, 60% of respondents stated that they were at least slightly annoyed by neighbour noise [15], making it the second most annoying noise source after road traffic noise [15].

These findings highlight the importance of tackling neighbour noise annoyance. However, the investigation of neighbour noise and related noise annoyance is a challenging area of research for several reasons.

Neighbour noise has heterogenous acoustical characteristics and sources, making it difficult to gather precise and reliable exposition data [16]. Neighbour noise covers a wide range of sounds, such as music from neighbours, footsteps, or sounds of tools, with an often irregular frequency. Additionally, neighbour noise is high in information content that can capture human attention [10].

The classification of noise as neighbour noise in judicial terms can differ from residents’ interpretation of neighbour noise. While the judicial definition of neighbour noise excludes, e.g., sounds originating from nearby restaurants or other food service industries, the sounds of a group of guests in a restaurant downstairs might be perceived as neighbour noise by a tenant. Thus, the definition of neighbour noise is rather ambiguous for lay people.

Furthermore, there are various factors that relate to annoyance ratings for neighbour noise. These include contextual (social and physical) and personal factors. Physical factors encompass aspects such as the agglomeration density of the area or the house type. Social aspects relate to social relations such as relationships to neighbours. Personal factors can be dispositions such as noise sensitivity or attitudes towards the source or towards noise polluters. Few studies have investigated the impact of these factors on neighbour noise annoyance, and some differences in relation to these factors have been identified [17,18,19,20,21,22,23].

Studies show that housing conditions seem to be linked to neighbour noise annoyance. With respect to the density of agglomeration, people living in rural areas experienced lower levels of neighbour noise annoyance than people living in areas with a higher density of agglomeration; e.g., [18,19]. In particular, the degree of neighbour noise annoyance was higher in people living in metropolitan areas [17]. In line with this, differences in annoyance ratings were observed depending on the type of house in which people live. Annoyance due to neighbour noise was more frequently reported by people living in multi-storey buildings in comparison to people living in detached houses [17,18,19]. The factor ownership status is linked to the housing situation. Tenants have been shown to be more likely to report neighbour noise annoyance than home-owners [18,19,20].

Another factor to consider with respect to neighbour noise annoyance is the time of the day at which noise occurs; in line with research on other noise sources, neighbour noise seems to be especially annoying during the nighttime [21].

Supported by research on other environmental noise sources [22], people’s attitudes towards the source of noise were also linked to neighbour noise annoyance ratings. When noise was perceived as unacceptable or caused by a negatively viewed neighbour, it was considered a reason for annoyance [21]. Neighbourhood conflicts were often associated with annoyance and could not be attributed solely to the exposure [20,23]. The relationship to one’s neighbours does not only impact noise annoyance but could also have an effect on mental health directly [24]. In [24], it was found that the relationship with one’s neighbours could, among other factors, influence psychological distress levels. Participants who reported being more satisfied with the relationship to their neighbours experienced less psychological distress compared to those who were less satisfied or unsatisfied with the relationship to their neighbours.

Finally, sociodemographic factors are linked to neighbour noise annoyance. Research shows that older people are less frequently annoyed by neighbour noise than younger people [17,18,19]. Moreover, a lower socio-economic status is linked to higher levels of noise annoyance due to neighbours [5], which might reflect the fact that people with socioeconomic disadvantages tend to live in multi-storey buildings.

New research regarding neighbour noise annoyance emerged during the COVID-19 pandemic; e.g., [24,25]. During the COVID-19 pandemic in 2020 and 2021, numerous countries imposed lockdowns to control the spread of the virus. In many countries, this led to a halt of public life; people had to stay at home, and many worked from home as well. Several studies examined the impact of the COVID-19 lockdowns in 2020 on people’s perception and experience of neighbour noise [24,25,26,27,28].

In one study [26], participants heard fewer neighbourhood sounds during lockdown than in the pre-lockdown phase (46% compared to 60% answering 3) “moderately”, 4) “very”, and 5) “extremely” on a five-point Likert scale). In line with this, fewer participants were annoyed by neighbour noise during the lockdown.

In [25], tweets were analyzed and a survey was conducted comparing people’s attitudes towards outdoor and neighbour noise before the COVID-19 pandemic and during the COVID-19 lockdown. The authors found more than twice as many noise complaints during the lockdown compared to 2019; specifically, complaints with respect to other people talking or shouting and to TV/music activities increased. The results of the survey reveal a decrease in the perceived outdoor noise levels and an increase in perceived neighbour noise levels.

In another study [27], 14% fewer noise complaints were found during the COVID-19 period (March to December 2020) in Dallas, USA, than in the same period in 2019. The reduction of complaints was observed in and around the city centre, but not in the outskirt areas. A descriptive analysis reveals that the content of these complaints changed between 2019 and 2020. In 2020, the most frequent noise complaints were about apartments, the neighbours and screaming, reflecting that people spent more time at their homes.

In [28], the authors assessed conflicts between neighbours during the COVID-19 lockdown in 70 Mexican cities. They compared the time periods of March and September 2020 with March and September 2019, respectively. Participants were asked whether there was any conflict with their neighbours in the past three months. A conflict with neighbours due to noise included noise from handicrafts (hammer blows, use of the drill) and loud music and parties. The authors found that noise-related conflicts increased by 42% during the lockdown. Population density and the type of home (apartment or house) were not significantly related to neighbour conflicts.

Neighbour noise annoyance seems to vary depending on different social, physical, and personal factors. However, it is still unclear which are the most important factors and which are the factors that neighbour noise annoyance is most strongly determined by.

The aim of the current study is to examine the role of different factors in contributing to annoyance from neighbour noise. It is hypothesized that several physical, social, and personal factors at least partly predict annoyance due to neighbour noise; in particular, we investigate for the first time the effect of the relationship to neighbours as a potential predictor of neighbour noise annoyance. The study was conducted as part of a representative survey on noise annoyance from different environmental noise sources in Germany. Note that the study was conducted in 2018 and 2019; i.e., the (increased) neighbour noise annoyance during the COVID-19 lockdown could not be studied in this survey. The noise sources studied were air traffic, road traffic, rail traffic, industry, wind turbine noise, and neighbour noise. The study was carried out in four different areas in Germany divided in sub-areas with different agglomeration densities to reflect the population density structure. Focus groups were conducted to gather first insights into the general understanding of neighbour noise in the population and important related factors. Based on these results, a questionnaire was developed. A multi method (online and paper–pencil) survey was conducted.

## 2. Materials and Methods

### 2.1. Study Design

Four study areas were selected in the north, east, west, and south of Germany in the greater areas of Hamburg, Dresden, Stuttgart, and Dusseldorf, respectively, to ensure a population-representative sample. Furthermore, the study areas were selected in such a way that they were representative of settlement areas with different degrees of density. In addition, the study areas were intended to reflect the typical noise pollution in Germany. Each area was divided into three agglomeration types: the inner-city area, the urban outskirt, and the rural area. The agglomeration types were based on national statistics of the population density and estimations of the population per km^2^ as depicted in [29]. For the definition of the three categories of agglomeration density, a ratio for the number of participants was applied according to the ratio of the population density of each area. As an example, in the study area of Hamburg, the ratio of the inner city, outskirt, and rural area was 4:2:1. Only around Dusseldorf was a ratio of 6:3:1 applied, as the region is less rural.

Selection criteria for the study areas included the existence of an airport in the area, the presence of a metropolitan area, and further availability of EU noise mapping results for road, rail, and air traffic, among others.

### 2.2. Procedure

In total, two survey waves were conducted addressing different random samples from the four study areas. Two survey modes were offered: an online survey and a paper–pencil version. A first study wave was conducted in 2018. Residents living in the selected study areas were randomly selected, received a cover letter including information about the study, and were invited to participate in the online survey. Due to a low response, a second study wave was conducted in 2019 with the additional option to participate by completing a paper–pencil questionnaire. The paper–pencil version of the questionnaire was enclosed together with a stamped envelope.

The survey was preceded by focus groups in two regions of Germany: one in an urban area in North Rhine Westphalia, and the other in a rural area in Bavaria. Both focus groups did not take place in the study areas of the main survey to avoid informing people about the study in the selected areas beforehand. The aim of the focus group was to get more detailed and qualitative information on people’s concepts of neighbourhood and environmental noise in general and neighbour noise in particular. This information was used to formulate questions referring to neighbourhood relations and sources of neighbour noise. For more information about the focus group results, see [16].

### 2.3. Variables

The questionnaire encompassed questions on the topics of the living environment, annoyance due to different noise sources (road, rail, air, neighbourhood, industry, sports grounds and leisure facilities), sleep disturbances due to different noise sources, personal factors, and sociodemographics. Some questionnaire items were taken from previous socio-acoustic surveys; e.g., [30,31,32]. Others, specifically those referring to the neighbourhood and to neighbour noise, were developed for this study based on a literature analysis and the focus group discussions preceding the survey. For the analyses in this study, the following variables were used:

Noise annoyance was assessed using the five-point verbal annoyance scale as recommended by the International Commission on Biological Effects of Noise (ICBEN) [33] and as specified in ISO/TS 15666 in 2003 [34], which was updated in May 2021 [35]. Besides annoyance due to neighbour noise, we assessed the annoyance due to road traffic as the most widespread source of environmental noise annoyance [15], followed by noise due to the food service industry. The latter source was assessed because focus group participants often included noise from (open-air) pubs, restaurants, or from people on the street leaving these establishments in their definition of neighbour noise, even though gastronomy locations belong to the source “industry and trade” from a legal point of view.

The type of house that participants lived in was assessed using the following categories: detached house, end-terrace house, mid-terrace house, semi-detached house, and apartment in a multi-storey building. This categorization of house types was used in previous noise effect studies (e.g., [36]) and found to be useful for this study, as the categorization reflected the density of neighbours living together and potentially affecting exposure and responses to neighbour noise.

Residential satisfaction was assessed with the question “How satisfied are you with your living environment?”, which was answered on a five-point scale from (1) “not satisfied” to (5) “very satisfied”. Previous studies have shown that residential satisfaction is associated with noise annoyance [37], particularly when the question refers to the residential area outside the dwelling and not to the dwelling itself [38]. As a result of the focus groups, it was found that the connotation of the German term “living environment” (*Wohnumgebung*) refers more to spatial issues, whereas the connotation of the German term “neighbourhood” (*Nachbarschaft*) refers more to social issues; i.e., the relation between neighbours. To capture both connotations in the residential satisfaction assessment, we added two further questions: “How satisfied are you—all in all—with your neighbourhood?”, which was assessed on a five-point scale from (1) not satisfied to (5) very satisfied; and “All in all, how would you describe your relationship with your neighbours in general?”, with five answer categories from (1) very bad to (5) very good. Further, participants were asked if they perceived themselves as a source of neighbour noise (yes/no).

It is well known that, besides noise exposure, which could not be assessed for neighbour noise in this study, other, non-acoustic factors, contribute to noise annoyance [22,39,40]. There is a huge body of evidence that noise sensitivity modifies the exposure–response relationship of noise annoyance and further noise health effects; e.g., [41,42,43,44,45]. “Noise sensitivity refers to the internal states […] of any individual which increase their degree of reactivity to noise in general.” (p. 59, [41]). There are several standardized inventories assessing noise sensitivity. However, to reduce the time that participants needed to answer the questionnaire, noise sensitivity was assessed in terms of a self-reported single item: “How sensitive are you to noise in general?”, with a five-point answering scale from (1) not sensitive to (5) very sensitive. Further, socio-demographics were assessed, including gender and age. From these socio-demographic variables, age was included in the analysis, as age is known to have an inverted U-shaped relationship with annoyance; i.e., middle-aged adults (about 40 to 60 age) are more annoyed than younger and older people [46]. For a more detailed description of the methodology and the questionnaire, see [16].

### 2.4. Analysis

Descriptive data analysis was performed in terms of the calculation of frequencies, means, and standard deviations. The distribution of characteristics was analyzed depending on levels of neighbour noise annoyance, with Chi-square tests for categorial variables, Cramers φ for effect size, and F-Tests/ANOVAs for continuous variables. Correlation analysis was performed to examine the relationship between variables. When Pearson’s correlation coefficient was *r* > +/− 0.2, the variables were selected for the regression analyses. To analyze the impact of several variables on the manifestation of annoyance due to neighbour noise, linear regression models were performed using generalized linear models (GZLM) with the predictors of age, density of agglomeration, residential satisfaction, relationship to neighbours, satisfaction with neighbourhood, annoyance due to other noise sources (road, food service industry), and noise sensitivity. The GZLM procedure allows the application of robust estimators (Huber–White sandwich estimators; [47,48]), which provide consistent covariance estimates even in the presence of assumption violations on the distribution shape and variance homogeneity.

A *p*-value <0.05 was considered significant. The statistical analyses were carried out using IBM SPSS Statistics, version 27.0 (IBM Deutschland GmbH, Ehningen, Germany).

## 3. Results

First preliminary results were presented at the 13th ICBEN Congress on Noise as a Public Health Problem 2021 [49]. In this paper, we discuss the role of factors contributing to the prediction of noise in more detail.

### Descriptive Statistics

The sample consisted of 1973 participants with 50.5% online participation. For the current analyses, 33 participants were excluded due to missing data on the noise annoyance items. Thus, 1940 respondents were considered for further analyses. The mean age was 57.1 years (*SD* = 14.36; age ranges from 18 to 94), with 55% female participants.

Most participants lived in the inner city (62 %) and in apartments in multi-storey buildings (79%). Most participants were tenants. Residential satisfaction was, on average, quite high, with a mean of 4.0 (*SD* = 0.9). Generally, participants rated their relationship to neighbours as good (*M*= 3.9; *SD* = 0.7) and were very satisfied with their neighbourhood (*M*= 4.0; *SD* = 0.8).

Overall, participants were slightly annoyed by neighbour noise (*M* = 2.03; *SD* = 0.95). The highest annoyance due to a specific noise source was reported for road traffic (*M*= 2.4; *SD* = 1.2). Participants reported an average noise sensitivity of 2.8 (*SD* = 1.0). Approximately two thirds of participants did not perceive themselves as being a noise polluter.

An overview of the descriptions for the sample as well as the descriptive statistics for each level of neighbour noise annoyance is shown in Table 1 and Table 2.

Chi-square tests and ANOVAs revealed significant differences between various characteristics such as the density of the agglomeration (χ^2^(8) = 32.20, *p* < 0.001, φ = 0.09) and different levels of neighbour noise annoyance (See Table 1 and Table 2). For example, the mean age significantly differed between different levels of neighbour noise annoyance, with the mean age decreasing with higher annoyance levels (F(4,1907) = 21.796, *p* < 0.001). Additionally, there were significant differences between levels of neighbour noise annoyance with respect to residential satisfaction (F(4,1905) = 71.376, *p* < 0.001), relationship with neighbours (F(4,1928) = 70.968, *p* < 0.001), and satisfaction with the neighbourhood (F(4,1928) = 111.981, *p* < 0.001). For all three variables, the mean score decreased with increasing neighbour noise annoyance. For noise sensitivity, the mean scores tended to increase with higher neighbour noise annoyance levels, indicating that people who were more noise-sensitive experienced higher noise annoyance due to neighbour noise. The ANOVA revealed a significant difference between annoyance levels and noise sensitivity (F(4,1911) = 32.93, *p* < 0.001). Further, noise annoyance levels due to other noise sources seemed to differ between neighbour noise annoyance levels; e.g., road traffic noise annoyance (F(4,1859) = 40.995, *p* < 0.001), food service industry (F(4,1867) = 27.08, *p* < 0.001), and industry (F(4,1883) = 19.15, *p* < 0.001).

Correlations were calculated to select suited predictors of annoyance due to neighbour noise (See Table 3). As a cut-off, a correlation of *r* > +/− 0.2 was chosen. The results show that neighbour noise annoyance was significantly correlated with all other variables (see Table 3). For example, neighbour noise annoyance was strongly negatively correlated with satisfaction with the neighbourhood (*r* = −0.43), relationship with neighbours (*r* = −0.35), and residential satisfaction (*r* = −0.35). Annoyance due to road traffic noise (*r* = 0.28) and due to noise from the food industry (*r* = 0.23) showed a highly significant correlation with neighbour noise annoyance as well. Age was negatively linked to neighbour noise annoyance (*r* = −0.20). Based on the correlation results and previous studies, seven variables were selected as potential predictors of neighbour noise annoyance and included in further analyses.

**Figure 1:**
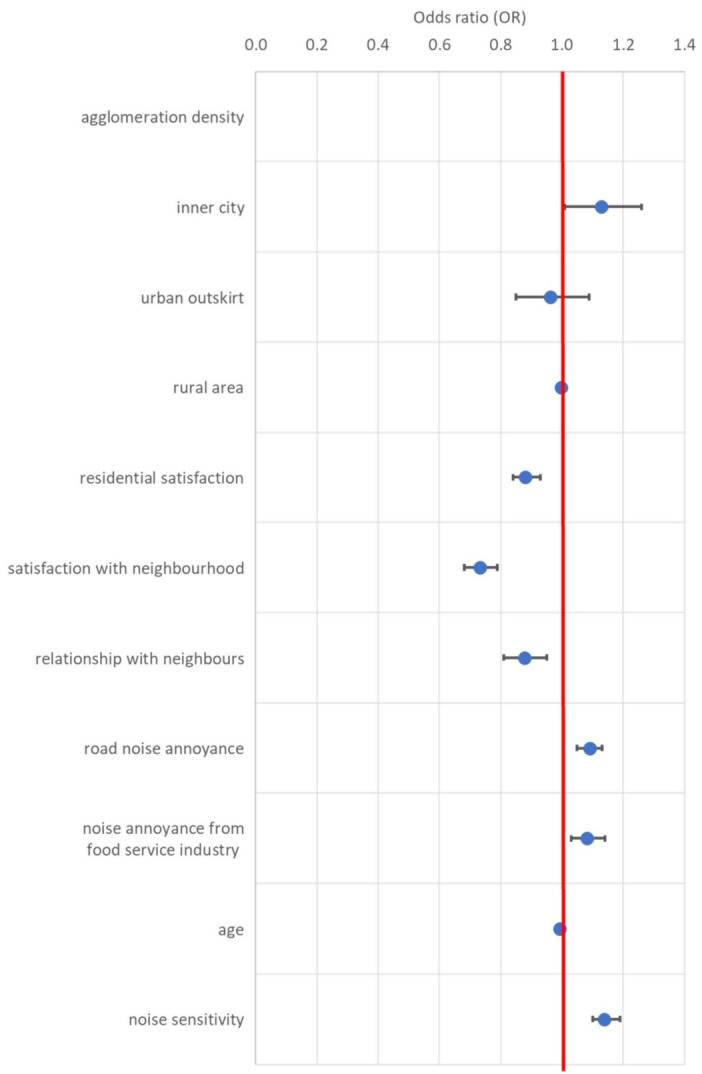
Odds ratios (OR) of the predictors to neighbour noise annoyance derived from the GZLM regression analysis.

A Generalized Linear Model with neighbour noise annoyance as the dependent variable was run to assess the predictive value of the independent variables on neighbour noise annoyance. Table 4 shows the regression results. The odds ratios of the predictors of neighbour noise annoyance are depicted in Figure 1.

As agglomeration density is a categorical variable, the category rural area served as a reference group. The regression results indicate that living in the inner city significantly increased the probability of being annoyed by neighbour noise (OR = 1.13, CI (1.01,1.26)), whereas living in the urban outskirt did not (OR = 0.96, CI (0.85,1.09)).

The continuous predictors in the model all reached significance. The odds of being annoyed by neighbour noise were 1.09 times greater for each 1-point increase in annoyance due to road traffic noise as well as noise annoyance due to the food service industry.

In contrast, the probability of being annoyed by neighbour noise was lower for participants with higher residential satisfaction (OR = 0.88; CI(0.84,0.93)), a higher satisfaction with their neighbourhood (OR = 0.73; CI(0.68,0.79)), and a better relationship with neighbours (OR = 0.88; CI(0.81,0.95)). The odds ratios for age and noise sensitivity were 0.99 (CI(0.99,1.00)) and 1.14 (CI(1.10,1.19)), respectively.

## 4. Discussion

The current study examined the influence of contextual and personal factors on neighbour noise annoyance.

Confirming the results of previous studies [17,18,19], the density of agglomeration was found to have an impact on neighbour noise annoyance. Living in the inner city was associated with higher neighbour noise annoyance ratings and seemed to increase the probability of being highly annoyed by neighbour noise compared to living in rural areas. However, the effects were relatively small. Inner cities are usually more densely populated and there are more multi-storey buildings than in rural areas. Thus, there are more people living in smaller spaces, which increases the number of potential noise sources to which a person is exposed. Thus, it is advised not to solely investigate the factor of the agglomeration area but to take other housing aspects into account in further studies as well.

Satisfaction with the neighbourhood and residential satisfaction both influenced neighbour noise annoyance. The strongest effect was found for satisfaction with the neighbourhood. These results suggest that both satisfaction measures seem to be important for the perception of neighbour noise. Being predominantly satisfied with the living environment or the neighbourhood—e.g., due to nearby green spaces, a good accessibility of facilities for daily needs, a good connection to public transport, etc.—could compensate for aspects such as neighbour noise, which may be more disturbing if one is generally not very satisfied with the living environment. Additionally, satisfying neighbourhoods might offer social cohesion and more options for recreation or respite to recover from neighbour noise, even if these neighbourhoods are noisier. Both the restorative quality of a neighbourhood and social cohesion are known to be associated with reduced road noise annoyance and to beneficially mediate the relationship between noise annoyance and mental health [50]. It can be assumed that this might be also true for neighbor noise. One could argue that satisfaction with the neighbourhood and residential satisfaction refer to the same concept. However, the difference in the magnitude of the influence of both variables on neighbour noise annoyance indicates that participants respond to conceptually different items. When asked about their residential satisfaction, participants might think about physical features and different aspects that their close environment has to offer (e.g., shopping possibilities, parks, etc.). In contrast, satisfaction with the neighbourhood might focus on the social aspect of one’s close environment; i.e., the social contact with other people living in the same area. As the current study used a cross-sectional design, no conclusions can be drawn about the causal pathway. There could be a reciprocal relationship between these three variables. Neighbour noise annoyance could influence residential satisfaction and satisfaction with the neighbourhood and vice versa.

In addition, the results indicate that the social relationship with people living in the surrounding neighbourhood seems to be relevant for how neighbour noise is perceived. A better relationship to neighbours is linked to lower neighbour noise annoyance. This corresponds to recent findings where neighbour noise annoyance was associated with conflicts with neighbours [17,20]. It further corresponds to findings that satisfaction with the relationship to neighbours was associated with the perception of psychological distress [24]. A noise-causing neighbour might lead to annoyance, which might in turn affect the relationship to that neighbour. A general positive feeling towards the person causing the noise might enhance the acceptance of that noise or decrease the perception of specific sounds as noise in the first place. The concept of the relationship to neighbours might be similar to the non-acoustic factor attitudes towards a noise source, which is known to impact noise annoyance levels. For example, positive attitudes towards a noise source are associated with less noise annoyance [31], whereas negative attitudes are linked to higher noise annoyance ratings [51]. The association of the relationship to neighbours and neighbour noise annoyance found in this study corresponds to the findings in [52]. The authors found that the type of relationship to one’s neighbours (e.g., friends or enemies) is linked to different types of coping strategies [52].

In line with other studies dealing with different noise sources [53,54], noise sensitivity was found to significantly influence neighbour noise annoyance.

Annoyance from other noise sources had a significant effect on neighbour noise annoyance as well, although the effect size was only small. Road traffic noise annoyance seems to be linked to neighbour noise annoyance, but participants differentiate between the two noise sources. The effect of annoyance due to food service industry noise on neighbour noise is significant, but also small. This might be due to the different densities of agglomeration in the study areas. There are probably more food services or restaurants located in the inner city than in rural areas. Future studies could specifically take this difference into account and investigate the effect of noise annoyance due to the food service industry on neighbour noise annoyance in inner cities.

The current study is restricted by several limitations that need to be considered in future research. First, the study was conducted with a cross-sectional design. Thus, it cannot provide information about the causal pathway between variables. Second, no sound exposure data could be assessed. As neighbour noise consists of a great variety of heterogenous sound elements (i.e., voices, slamming doors, lawn mower, etc.) and occurs at different times during the day, it is difficult to predict and model the noise, which indicates that sound measurements would be necessary for neighbour noise exposure assessment, which, again, was not part of the current study. Due to the lack of exposure data, the influence of the sound levels and acoustical characteristics of different neigbourhood noise sources remains unclear.

For example, people might be more annoyed by noise due to door slamming compared to music. In order to estimate the impact of sound pressure levels and acoustical characteristics on the noise annoyance judgement, future studies should include sound exposure and acoustical characteristics (for example, sharpness or informative value) and consider the time of day of exposure. Being able to differentiate between different subtypes of neighbour noise might also help to develop suitable interventions to prevent or address neighbour noise annoyance more precisely. Furthermore, differentiating between indoor and outdoor neighbour noise (explicit noise from indoor neighbours vs. the specific neighbourhood) could help to locate and specify the noise problem. Moreover, there seems to be a relevant social component of the relationship to neighbours influencing neighbour noise annoyance, which needs further consideration.

As there is a national trend to build more densely populated residential areas, especially in larger cities, to reduce daily individual car travel and use housing space more efficiently, neighbour noise annoyance is already relevant and will become an even more relevant environmental noise source to consider. Further research is needed to identify the factors that contribute to neighbour noise annoyance and integrate this knowledge in planning policies.

## 5. Conclusions

The current study assessed which factors contribute to the annoyance due to neighbour noise aside from noise levels by means of a cross-sectional study with 1973 participants. Results indicate moderate effects of residential satisfaction, satisfaction with the neighbourhood, and relationship to neighbours on neighbour noise annoyance. The risk for the perception of neighbour noise annoyance decreased with an increase in these factors. Considering that neighbour noise annoyance is the second most annoying noise source in Germany, and that a large number of cities in Germany are experiencing an increase in densely populated areas, neighbour noise should be considered in land-use and city planning.

Future research is required to investigate other potential contributing factors to noise annoyance. In future studies, it could be interesting to differentiate between different neighbour noise sources. Further, the use of different coping strategies depending on the relationship to the neighbours could be examined.

## Figures and Tables

**Table 1:** Descriptions by annoyance levels (five-point verbal scale levels).

				Annoyance due to Neighbour Noise						
	N		Sc	Not at all	Slightly	Moderately	Very	Extreme-ly	Total	Sign
Total	1940	100		31.9	43.2	16.9	6.1	2.0	100	
Age	1912		M	61.0	56.4	53.6	52.5	51.4	57.1	***
Gender										
Female	584	55	%	38.2	39.7	15.2	5.1	1.7	100	n.s.
Male	487	45	%	35.3	43.3	14.4	4.9	2.1	100	
Density of agglomeration										
Inner city	1207	62	%	27.8	43.7	18.8	7.1	2.5	100	***
Urban outskirt	436	22	%	39.0	42.5	13.4	3.8	1.4	100	
Rural area	307	16	%	36.4	43.0	13.9	5.6	1.0	100	
House type										
Detached house	151	8	%	36.4	49.0	10.6	2.0	2.0	100	**
End-terrace house	61	3	%	44.3	41.0	9.8	3.3	1.6	100	
Mid-terrace house	87	4	%	35.6	48.3	12.6	3.4	0.0	100	
Semi-detached house	84	4	%	41.7	45.2	7.1	4.8	1.2	100	
Apartment in multi-storey building	1532	79	%	29.9	42.7	18.3	6.9	2.2	100	
Ownership										***
Owner	762		%	32	48	15	4	1	100	
Tenant	1170		%	31	41	17	8	3	100	
One’s own perception as a causer of noise										
Yes	586		%	27	47	17	8	2	100	*
No	1295		%	34	42	17	6	2	100	

*** *p* < 0.001, ** *p* < 0.01, * *p* < 0.05 (χ^2^ for categorial variables, F for rating scales), N= number of participants, M = mean, Sc = scale, n.s. = not significant.

**Table 2:** Descriptions by annoyance levels (five-point verbal scale levels).

.		Annoyance due to Neighbour Noise						
	N	Not at all	Slightly	Moderately	Very	Extremely	Total	Sign
Residential satisfaction	1910	4.3(0.8)	4.1(0.8)	3.7(0.8)	3.3(1.1)	2.8(1.3)	4.0(0.9)	***
Relationship to neighbours	1933	4.2(0.6)	4.0(0.6)	3.7(0.7)	3.4(0.7)	3.2(0.8)	3.9(0.7)	***
Satisfaction with neighbourhood	1933	4.3(0.7)	4.0(0.7)	3.7(0.8)	3.2(0.9)	2.8(1.0)	4.0(0.8)	***
Noise annoyance living environment in general	1928	2.1(1.1)	2.6(1.0)	3.2(0.9)	3.7(0.8)	4.5(0.6)	2.7(1.1)	***
Road noise	1864	2.0(1.1)	2.4(1.1)	2.8(1.2)	3.0(1.3)	3.2(1.4)	2.4(1.2)	***
Rail noise	1818	1.3(0.7)	1.4(0.9)	1.6(1.1)	1.5(1.0)	1.8(1.2)	1.4(0.9)	***
Food service industry	1872	1.2(0.6)	1.3(0.7)	1.6(1.0)	1.8(1.1)	2.1(1.6)	1.4(0.8)	***
Industry	1888	1.1(0.4)	1.2(0.6)	1.4(0.8)	1.5(0.9)	1.6(1.2)	1.2(0.6)	***
Noise sensitivity	1916	2.5(1.0)	2.9(0.9)	3.1(1.0)	3.1(0.0)	3.7(1.0)	2.8(1.0)	***

*** *p* < 0.001 (χ^2^ for categorial variables, F for rating scales), N = number of participants, M = mean, Sc = scale.

**Table 3:** Product–moment correlations between potential contributors to neighbour noise annoyance.

	1	2	3	4	5	6	7	8	9	10
Neighbour noise annoyance	1									
Age	−0.20 ***	1								
Noise sensitivity	0.24 ***	−0.06 **								
Residential satisfaction	−0.35 ***	0.13 ***	−0.09 ***	1						
Satisfaction neighbourhood	−0.43 ***	0.14 ***	−0.06 *	0.44 ***	1					
Relationship neighbours	−0.36 ***	0.12 ***	−0.04	0.32 ***	0.71 ***	1				
Annoyance road	0.28 ***	−0.14 ***	0.08 ***	−0.42 ***	−0.20 ***	−0.14 ***	1			
Annoyance rail	0.14 ***	−0.09 ***	0.01	−0.19 ***	−0.12 ***	−0.09 ***	0.28 ***	1		
Annoyance food industry	0.23 ***	−0.09 ***	0.14 ***	−0.28 ***	−0.17 ***	−0.10 ***	0.25 ***	0.06 *	1	
Annoyance industry	0.20 ***	−0.06 *	0.03	−0.26 ***	−0.16 ***	−0.12 ***	0.21 ***	0.09 ***	0.23 ***	1

*** *p* < 0.001; ** *p* < 0.01; * *p* < 0.05.

**Table 4:** Results of GZLM regressions for the analysis of predictors of neighbour noise annoyance.

Predictor	B	*SE*	Wald	*p*	OR	CI −	CI +
Constant	3.865	0.1869	427.75	<0.001	47.690	33.06	68.78
Agglomeration density							
Inner city	0.122	0.0560	4.78	<0.05	1.130	1.01	1.26
Urban outskirt	−0.037	0.0649	0.32	0.570	0.964	0.85	1.09
Rural area	*Ref.						
Residential satisfaction	−0.126	0.0260	23.41	<0.001	0.882	0.84	0.93
Satisfaction with neighbourhood	−0.309	0.0359	74.07	<0.001	0.734	0.68	0.79
Relationship with neighbours	−0.127	0.0398	10.14	<0.001	0.881	0.81	0.95
Road noise annoyance	0.089	0.0185	22.79	<0.001	1.093	1.05	1.13
Noise annoyance from food service industry	0.082	0.0251	10.63	<0.001	1.085	1.03	1.14
Age	−0.006	0.0014	17.45	<0.01	0.994	0.99	1.00
Noise sensitivity	0.132	0.0201	42.86	<0.05	1.141	1.10	1.19

B = regression coefficient, SE = standard error, Wald = Wald Chi-square, *p* = significance level, OR = odds ratio, CI− = lower confidence interval, CI + = upper confidence interval, *Ref. = reference group.

## Data Availability

Data sharing not applicable.
